# O.3.1-3 Self-evaluation survey as a tool for planning, monitoring and evaluation physically active operating culture at school

**DOI:** 10.1093/eurpub/ckad133.140

**Published:** 2023-09-11

**Authors:** Katariina Kämppi, Harto Hakonen, Kati Lehtonen, Katja Rajala

**Affiliations:** Jamk University of Applied Sciences, Likes, Finland; katariina.kamppi@jamk.fi; Jamk University of Applied Sciences, Likes, Finland; katariina.kamppi@jamk.fi; Jamk University of Applied Sciences, Likes, Finland; katariina.kamppi@jamk.fi; Jamk University of Applied Sciences, Likes, Finland; katariina.kamppi@jamk.fi

## Abstract

**Purpose:**

Since 2010, Finland has been building a concept called Finnish Schools on the Move (FSM), which is a national action programme supporting educational institutions to develop a more physically active operating culture. The Self-Evaluation Survey for Physical Activity (PA) Promotion in School is a tool developed in 2015 for FSM to support schools in implementing their own unique action plans and to monitor the progress of the programme at the levels of schools, municipalities, and the nation.

**Methods:**

The online survey, available on the programme’s website, has altogether 36 statements. Teams in schools evaluate each statement using a scale of 0-4 (0=not at all, 4= fully realised). To summarise the results, four indexes have been formulated: strategy, facilities for PA, actions, and support. The scale was converted to a percentage of the maximum score. The strategy index describes plans and principles that lead the actions. The facilities index includes indoor and outdoor facilities and equipment. The actions index consists of all the actions that enable PA, and the support index consists of all the collaboration that supports actions.

**Results:**

By the end of February 2023, the data consisted of nearly 5,000 self-evaluations concerning the physically active operating culture. The newest index results (2022-23) show that the strategy (71.3%) and facilities (62.1%) achieved the highest values, and the lowest values were given to actions enabling PA (59.5%) and support (51.0%). The progress of the FSM programme can be examined using the index results over the past eight school years.

**Conclusions:**

The self-evaluation survey results are available online and are offered on three levels: national, regional, and municipal. At all these levels, the self-evaluation produces up-to-date information on the PA promotion progress in schools for PA promotors, coordinators, and decision-makers. For schools, it provides a diverse tool that increases schools’ awareness of the state of their physically active operating culture and inspires discussions among staff to find areas for development. By repeating the survey regularly every year, the progress of PA promotion in a school can be monitored in the results.

**Support/Funding Source:**

The Ministry of Education and Culture of Finland.

